# Effects of Water Avoidance Stress as a Psychological Stress Model and Coenzyme Q10 on Reproductive, Endocrine, and Ovarian Responses in Adult Female Rats

**DOI:** 10.3390/ani16132093

**Published:** 2026-07-06

**Authors:** Ahmet Yardimci, Tugrul Ertugrul, Ebru Gokdere, Feyza Keskin Buyukbudak, Meryem Sedef Dogru, Ahmet Tektemur, Zeliha Irem Turk, Nazife Ulker Ertugrul, Serife Tutuncu, Sinan Canpolat

**Affiliations:** 1 Department of Physiology, Faculty of Medicine, Firat University, 23119 Elazig, Turkey; 2 Department of Histology and Embryology, Faculty of Veterinary Medicine, Ondokuz Mayis University, 55200 Samsun, Turkey; 3 Experimental Research Center, Firat University, 23119 Elazig, Turkey; 4 Department of Medical Biology, Faculty of Medicine, Firat University, 23119 Elazig, Turkey; 5 Department of Physiology, Faculty of Medicine, Samsun University, 55080 Samsun, Turkey; 6 Department of Physiology, Faculty of Medicine, Dokuz Eylul University, 35220 Izmir, Turkey

**Keywords:** psychological stress, coenzyme q10, water avoidance stress, female reproductive function, sexual incentive motivation, reproductive hormones, 17-β estradiol, oxidative stress response, kisspeptin

## Abstract

Psychological stress can affect female reproductive function through behavioral, endocrine, ovarian, and oxidative mechanisms. Antioxidant supplements have therefore attracted attention for their potential to mitigate stress-related reproductive alterations. Coenzyme Q10 (CoQ10) is a lipid-soluble quinone involved in mitochondrial energy metabolism and is widely used as a dietary supplement. However, whether CoQ10 modulates female reproductive responses to repeated psychological stress remains unclear. Although water avoidance stress (WAS) is a well-established psychogenic stress model, its effects on female reproductive outcomes are still not fully defined. In this study, we examined how repeated WAS affects female reproductive outcomes and whether CoQ10 modifies these effects. Twenty-eight regularly cycling female rats were assigned to sham control, WAS, CoQ10, or WAS + CoQ10 groups. WAS was applied for 1 h/day for 10 days, and CoQ10 was administered orally at 100 mg/kg/day. Repeated WAS did not significantly alter sexual incentive motivation parameters, reproductive hormones, corticosterone, total antioxidant capacity (T-AOC), 8-hydroxy-deoxyguanosine (8-OHdG), or mast cell count under the present experimental conditions (all *p* > 0.05). However, WAS reduced male-directed active investigation time (*p* = 0.008) and male investigation preference ratio (*p* = 0.024), increased absolute ovarian and adrenal gland weights (*p* = 0.035 and *p* = 0.016, respectively), reduced primordial follicle number (*p* = 0.030), decreased germinative epithelium thickness (*p* = 0.017), lowered VEGF histoscore (*p* = 0.033) regardless of CoQ10 treatment, and reduced corpus luteum angiogenesis in animals not receiving CoQ10 (*p* = 0.030). CoQ10 reduced total investigation time toward the male (*p* = 0.032), male investigation preference ratio (*p* = 0.037), 17-β estradiol (E2) (*p* = 0.003), testosterone (*p* = 0.021), and germinative epithelium thickness (*p* < 0.001) regardless of WAS exposure. CoQ10 also decreased kisspeptin-1 levels under non-stressed conditions (*p* = 0.010), while increasing corpus luteum angiogenesis under stress conditions (*p* = 0.003). Overall, repeated WAS produced selective behavioral and ovarian alterations rather than broad reproductive dysfunction. CoQ10 was not associated with a broadly protective or uniformly beneficial profile in this model, and its endocrine, behavioral, and ovarian effects should be interpreted with caution.

## Simple Summary

Psychological stress may affect female reproductive health, including sexual behavior, hormone regulation, and ovarian function. Antioxidants are often studied because they may help reduce stress-related biological damage. Coenzyme Q10 (CoQ10) is a commonly used antioxidant supplement, but its effects on female reproduction under repeated psychological stress remain poorly understood. Water avoidance stress (WAS) is a rat model of psychological stress, but its effects on female reproductive outcomes are still not fully clear. In this study, we examined how repeated WAS affects female reproductive outcomes and whether CoQ10 modifies these effects. Repeated WAS was not associated with a broad impairment of female reproductive function. Sexual incentive motivation, reproductive hormones, and oxidative stress markers showed no statistically detectable changes in most measured endpoints. However, WAS reduced active investigation behavior toward the male stimulus, increased absolute ovarian and adrenal gland weights, and decreased some ovarian parameters, including primordial follicle number, germinative epithelium thickness, VEGF immunoreactivity, and corpus luteum angiogenesis. CoQ10 did not show a generally protective effect in this model. Instead, it produced mixed effects: it reduced active investigation behaviors, 17-β estradiol (E2), and testosterone regardless of stress exposure; decreased kisspeptin-1 under non-stressed conditions; and increased corpus luteum angiogenesis under stress conditions. Overall, repeated WAS did not cause broad reproductive impairment in this model; instead, it produced limited behavioral and ovarian alterations. CoQ10 did not behave as a simple protective supplement in this model; its endocrine, behavioral, and histomorphological effects do not support a favorable overall reproductive profile. Therefore, CoQ10 should be evaluated cautiously in stress-related female reproductive models rather than assumed to be protective.

## 1. Introduction

Repeated psychological stress is particularly relevant to female reproductive dysfunction because activation of the hypothalamus–pituitary–adrenal (HPA) axis can interfere with hypothalamus–pituitary–ovarian (HPO) axis regulation at multiple levels [1]. Stress-induced activation of the HPA axis increases corticotropin-releasing hormone (CRH) activity and glucocorticoid exposure, which may suppress the GnRH pulse generator and consequently disrupt downstream gonadotropin output [2,3,4,5]. Beyond these central hypothalamic–pituitary effects, elevated glucocorticoid exposure may also impair ovarian function at the peripheral level by altering steroidogenic capacity, follicular development, and ovulatory function [6]. Water avoidance stress (WAS) is a well-characterized rodent model of repeated psychogenic stress [7,8,9] and provides an experimental framework for investigating sustained stress-related neuroendocrine, behavioral, and peripheral tissue responses. In this context, WAS enables the assessment of how repeated psychological stress influences female reproductive function in adult rats. Consistent with this framework, accumulating evidence indicates that stress-related disruption of female reproductive function extends from ovarian structure and endocrine regulation to sexual behavior. Previous studies have demonstrated that stress disrupts the follicular development [10,11,12,13,14], impairs the developmental potential of oocytes [15,16,17], and may attenuate ovarian growth while elevating oxidative stress, loss of ovarian follicles [18], and apoptosis [19]. Moreover, stress decreases reproductive hormone levels, including E2, progesterone [14,20], gonadotropin-releasing hormone (GnRH) [21], kisspeptin [22], and testosterone [23].

Additionally, stress may adversely impact the sexual behavioral aspect of female reproductive function. Clinical studies have reported decreased sexual desire in women with stress [24,25]. Because sexual desire in humans is conceptually comparable to the appetitive phase of sexual behavior in female rats, assessing sexual motivation in female rats may inform translational applications in the motivational aspects of women’s sexuality [26,27]. However, women’s sexual desire is highly complex, and it includes biological, psychological, social, and contextual components [28]; therefore, sexual incentive motivation in female rats cannot reproduce this broader human complexity. Nevertheless, animal models are beneficial for predicting physiological changes in the sexual behavioral dimension of female reproductive function, specifically the motivational components of female sexual behavior, including sexual desire. In animal models, stress has been associated with impairments in the appetitive [29] and receptive components of female rat sexual behavior [30,31].

Beyond its effects on folliculogenesis, endocrine balance, and reproductive behavior, stress also modifies the ovarian microenvironment. This microenvironment comprises stromal, vascular, and immune-related components that jointly support follicular development, ovulation, and corpus luteum formation [32]. Among the immune-related components, mast cells (MCs) are particularly relevant because stress-related mediators, including, CRH, can activate MCs and promote the release of vasoactive mediators, including vascular endothelial growth factor (VEGF) [33,34]. Moreover, stress may increase the number of MCs in various tissues including the female reproductive system [35,36,37]. These mast-cell-related responses may intersect with the vascular component of the ovarian microenvironment, in which VEGF is essential for angiogenesis and pivotal to follicular development and corpus luteum formation [38]. Moreover, it is considered a primary angiogenic factor contributing to luteal vascularization after ovulation [39]. VEGF, a major regulator of ovarian angiogenesis, was therefore evaluated in combination with MCs to examine the vascular–immune interface of stress-associated changes in the ovarian microenvironment. Stress-associated ovarian and reproductive alterations have been reported across various stress paradigms, including heat stress, cold stress, restraint stress, chronic unpredictable mild stress (CUMS), and combined forced swimming plus restraint stress [10,14,16,23,40]. By contrast, WAS studies have largely focused on colonic responses, visceral hypersensitivity, urinary bladder dysfunction, and mucosal immune activation [9,41,42], whereas the potential impact of WAS on female reproductive function remains insufficiently characterized. In particular, it is not known whether chronic WAS disrupts the motivational component of female sexual behavior together with reproductive hormone profiles, ovarian follicular morphology, and local ovarian VEGF and MC responses.

Accumulating experimental evidence suggests that stress-induced reproductive dysfunction is closely associated with oxidative stress, mitochondrial impairment, apoptosis, and altered ovarian function [43,44,45,46]. Therefore, antioxidant interventions have been investigated as potential modulators of stress-associated ovarian alterations. Within this framework, coenzyme Q10 (CoQ10), or ubiquinone, is a lipid-soluble endogenous quinone with antioxidant and cytoprotective properties that has received increased attention in female reproductive research [47,48,49]. CoQ10 is an integral component of the inner mitochondrial membrane, where it is central to electron transfer within the mitochondrial respiratory chain during oxidative phosphorylation, ultimately yielding adenosine triphosphate [50]. Although direct evidence regarding CoQ10 treatment in psychogenic stress-induced reproductive dysfunction remains limited, previous studies have examined the therapeutic effects of CoQ10 in ovarian aging [48], diminished ovarian reserve [47,51], and toxic damage [49]. However, limited knowledge exists regarding whether CoQ10 can modulate ovarian responses under repeated psychogenic stress, particularly in WAS.

Here, we hypothesized that repeated WAS would impair female reproductive function at behavioral, endocrine, and ovarian levels and that concurrent CoQ10 treatment would attenuate these stress-induced alterations. More specifically, WAS was expected to reduce sexual incentive motivation, disrupt reproductive hormone balance, and induce adverse ovarian changes involving oxidative, histopathological, angiogenic, immune–cellular, and molecular components. To test these hypotheses, we performed a comprehensive behavioral, endocrine, histological, and molecular assessment of female reproductive function and the ovarian microenvironment.

## 2. Materials and Methods

### 2.1. Sample Size Determination

The sample size was calculated a priori for a two-way ANOVA corresponding to the 2 × 2 factorial design of the study using G*Power software (version 3.1, Heinrich-Heine-Universität Düsseldorf, Germany). The analysis was performed with an effect size of f = 0.60, an α level of 0.05, a power of 0.85, and four experimental groups. Based on these parameters, the minimum required total sample size was determined to be 28 animals, with 7 animals allocated to each experimental group. Because this calculation was based on a large effect size, the study was primarily powered to detect large effects; therefore, non-significant findings should not be interpreted as definitive evidence of no effect, particularly for small or moderate effects.

### 2.2. Animals

The Experimental Research Unit of Firat University (Elazig, Turkey) provided twenty-eight female Sprague-Dawley rats (≈10–12 weeks old and 250–300 g) exhibiting regular estrous cycles. All rats were housed under standard hygienic laboratory conditions with a reversed 12 h light/12 h dark cycle (lights off at 8:00 AM), a constant temperature of 21 ± 1 °C, and a relative humidity of 55 ± 5%. They were housed in Plexiglas cages in groups of three or four and had *ad libitum* access to food and water. Animals were randomly assigned to four groups: sham control, WAS, CoQ10, and WAS + CoQ10, with seven rats per group. These groups corresponded to a 2 × 2 factorial design with two factors: stress exposure (no WAS vs. WAS) and CoQ10 treatment (no CoQ10 vs. CoQ10). Accordingly, the sham control group represented no WAS/no CoQ10, the WAS group represented WAS/no CoQ10, the CoQ10 group represented no WAS/CoQ10, and the WAS + CoQ10 group represented WAS/CoQ10.

### 2.3. Estrous Cyclicity

After group allocation and before the start of the experimental procedures, animals in all groups were monitored daily with vaginal smears to ensure normal cyclicity. Vaginal smears were collected daily for approximately 8–10 days to confirm two consecutive regular 4–5-day estrous cycles. The smears were evaluated by an experienced investigator blinded to group assignment, and only rats exhibiting this criterion were included in the study [52]. The vaginal smear procedure was conducted as previously described in our earlier study [53].

### 2.4. Water Avoidance Stress (WAS) Protocol

The WAS model protocol was adapted from a previous study [54]. After the confirmation of regular estrous cyclicity, animals in WAS and WAS + CoQ10 groups were placed individually on a platform (6 × 6 × 6 cm, length × width × height) positioned at the center of a water-filled tank (25 °C), (43 × 26 × 19 cm, length × width × height) for 1 h daily over ten consecutive days at room temperature from 10:30 to 11:30 AM. The water level was maintained at approximately 1 cm below the platform. In the sham control group, rats were similarly placed on a platform at the center of an empty tank with the same properties. At the end of the WAS procedure, the animals were returned to their home cages. Fecal pellet output was not included as an outcome measure because the study was designed to investigate reproductive, endocrine, and ovarian effects of repeated psychogenic stress rather than autonomic or visceral stress responses.

### 2.5. CoQ10 Treatment

Animals in the CoQ10 (*n* = 7) and WAS + CoQ10 (*n* = 7) groups received CoQ10 (Solgar, 200 mg; Leonia, NJ, USA) [55,56] at a dose of 100 mg/kg/day dissolved in olive oil, which served as the vehicle, by oral gavage [57,58] for ten consecutive days after the daily WAS protocol [59,60]. Moreover, olive oil (1 mL/kg/day) via oral gavage was administered to the sham control group for ten days. The CoQ10 dose was selected based on a previous study in female rats showing that the same oral dose of (100 mg/kg/day, p.o.) CoQ10 exerted beneficial effects on ovarian tissue [61]. Therefore, 100 mg/kg/day oral CoQ10 was considered a dose with previously reported biological activity in the female reproductive system. In the present study, the duration of CoQ10 administration was adapted to the 10-day WAS protocol, and CoQ10 was administered throughout the 10-day experimental period to evaluate its potential effects on WAS-induced behavioral, endocrine, and ovarian alterations.

### 2.6. Sexual Incentive Motivation (SIM) Test

#### 2.6.1. SIM Box

The apparatus was a transparent rectangular Plexiglas box with three compartments (91.44 × 31.75 × 40 cm), as in our previous study [53]. Perforated Plexiglas partitions with 1 cm diameter holes separated the central test compartment from two smaller side compartments (each 16.51 cm long). On the SIM test day, as explained in more detail below, a female rat from each group was placed in the central compartment, while sexual- and social-stimulus animals were placed individually in the side compartments. Although this design prevented direct physical contact, it permitted visual, olfactory, and auditory interaction between the experimental female and the stimulus animals. A dashed black line drawn at the midpoint of the central compartment was used to determine the activity of the experimental female rats.

#### 2.6.2. Acclimation

For acclimation, one week before the SIM or active investigation test, animals were individually placed in the central compartment of the SIM apparatus twice and allowed to explore freely for 15 min without sexual or social stimulus animals [62,63]. The sexual stimulus animal was a sexually experienced, gonadally intact male rat, whereas the social stimulus animal was an ovariectomized (OVX) adult female rat. During the same acclimation period, each stimulus animal was placed individually in its respective compartment twice and allowed to acclimate for 15 min without the experimental female rat [62,63].

#### 2.6.3. SIM Test

At the end of the ten-day WAS and/or CoQ10 treatment period, the SIM test and active investigation test were performed 24 h after the final WAS session to reduce the effects of the immediate acute response to the final stress exposure and to evaluate the behavioral consequences of repeated WAS [64]. Before behavioral testing, the estrous cycle stage was determined by vaginal smear cytology, and only females confirmed to be in behavioral estrus (defined here as the estrus stage of the estrous cycle) were tested [63]. First, each animal in all groups was placed in the central compartment and allowed to adapt and move freely for 5 min. Afterward, the sexual and social stimulus animals were placed in their respective compartments, and the test recording was started. During this period, the time spent near the sexual or social stimulus animals was recorded for 10 min. When all four paws of the female were within the 12.7 cm area adjacent to the partition separating the stimuli, the rat was considered to be spending time next to the sexual or social stimulus. Accordingly, the time spent near the male rat (TWM), the time spent near the female rat (TWF), total social time (TST; defined as the sum of TWM and TWF), and the male preference ratio (calculated as TWM/TST) were determined. Furthermore, the activity was assessed as the total number of crossings through the center of the test apparatus over 10 min and was scored manually using a hand counter [65].

#### 2.6.4. Active Investigation Test

For all animals, active investigation behaviors were assessed concurrently with the SIM test in the same apparatus, as described by Hawcock et al. (2010) [66]. Active investigation is characterized by experimental female behaviors directed toward the barriers separating her from the stimulus animals, including sniffing, licking, chewing, and climbing, in an apparent attempt to reach the sexual or social stimulus. Accordingly, the duration of investigation directed toward the male rat (TIM), the duration of investigation directed toward the female rat (TIF), total investigation time (TIT; TIM + TIF), and the male investigation preference ratio (TIM/TIT) were recorded during the 10 min test. Video recordings of both the SIM and the active investigation were subsequently analyzed manually, using a stopwatch, by an experienced observer blinded to the experimental groups.

### 2.7. Termination of Experiments

After completing the SIM test and active investigation test, animals were euthanized by rapid decapitation without prior anesthesia, and tissue samples were collected. Since all animals were confirmed to be in estrus stage before behavioral testing, tissue collection was performed at the same estrous stage across all groups, thereby minimizing cycle-related variability in endocrine and ovarian outcomes [67]. Blood samples were obtained and centrifuged at 4000 rpm for 5 min at 4 °C to collect serum. Serum samples were stored at −20 °C for approximately 15 days until enzyme-linked immunosorbent assay (ELISA) analysis. Repeated freeze–thaw cycles were avoided. Additionally, the ovaries, uterus, and adrenal glands were collected to determine absolute organ weights. Relative organ weight (RW) was also calculated for each tissue and expressed as mg/100 g final body weight using the following formula: RW = (organ weight/body weight × 100) [68].

### 2.8. ELISA Analyses

Serum levels of kisspeptin-1 (YLA0433RA, YL Biont, Shanghai, China), GnRH (YLA0012RA, YL Biont, Shanghai, China), total antioxidant capacity (T-AOC) (YLA0311RA, YL Biont, Shanghai, China), 8-hydroxy-deoxyguanosine (8-OHdG) (YLA0061RA, YL Biont, Shanghai, China), 17-β estradiol (E2) (201-11-0177, SunRed, Shanghai, China), progesterone (201-11-0742, SunRed, Shanghai, China), testosterone (201-11-5126, SunRed, Shanghai, China), and corticosterone (201-11-0497, SunRed, Shanghai, China) were determined in accordance with the manufacturer’s instructions using a Multiskan FC microplate reader (Thermo Fisher Scientific, Waltham, MA, USA). Serum samples were analyzed without additional dilution and were not measured in duplicate; absorbance values were confirmed to be within the standard curve range.

### 2.9. RNA Extraction and RT-qPCR

Total RNA was extracted from rat ovary using a TRIzol-based method (ZT-LIZ-100, Softec, Türkiye), as previously described [69]. A microspectrophotometer (Nano400A, Allsheng, Hangzhou, China) was used to determine RNA concentration and purity, and samples with A260/A280 ratios of 1.8–2.2 were used for subsequent analyses. First-strand cDNA was synthesized from 1 µg of total RNA using a commercial reverse transcription kit including DNase I treatment (ServiceBio, Wuhan, China), according to the manufacturer’s instructions. Reverse transcription reactions were conducted in a thermal cycler (Veriti, Applied Biosystems, Singapore), and the resulting cDNA samples were stored at −20 °C until subsequent use. Quantitative real-time PCR (RT-qPCR) was carried out using a SYBR Green-based detection system (Blastaq 2× qPCR Master Mix, ABM, Richmond, BC, Canada) on an Applied Biosystems 7500 Real-Time PCR system (Applied Biosystems, Singapore). Each reaction was performed in a final volume of 10 µL, using a reaction mixture prepared according to the manufacturer’s instructions for the SYBR Green master mix. Table 1 lists gene-specific primers used in our study. Amplifications were performed in 96-well plates with three technical replicates per sample. Thermal cycling conditions included the initial denaturation at 95 °C for 3 min, followed by 40 cycles of denaturation at 95 °C for 15 s and annealing/extension at 60 °C for 1 min. A melt curve analysis was performed at the end of each run to verify amplification specificity, and a single peak was observed for each target gene. Relative gene expression levels were calculated using the 2^−ΔΔCt^ method. For normalization, the reference gene GAPDH was selected based on its stable expression across all experimental groups (Ct range: 15–25; variation < ±1 Ct). No-template controls (NTCs) were included in each run to exclude contamination. Primer efficiency was within the acceptable range (90–110%) for all targets.

### 2.10. Histological Analyses

For histological and immunohistochemical analyses, ovaries were excised and collected, then fixed in 10% formalin. Ovarian tissues were processed using standard histological procedures, dehydrated, and embedded in paraffin blocks. From each block, five serial cross-sections (5 µm thick) were obtained at 30 µm intervals. Following mounting, the sections were stained with hematoxylin and eosin (H&E), Crossman’s triple stain, toluidine blue, and periodic acid–Schiff (PAS). H&E staining was used to evaluate germinative epithelium thickness and corpus luteum angiogenesis; Crossman’s triple staining was employed for follicular evaluation; toluidine blue staining was used for MC counting; and PAS staining was used to determine zona pellucida reactivity.

All sections were examined under 10× magnification and photographed using a microscope (Nikon Eclipse 50i, Tokyo, Japan). For each animal, histological assessments were conducted across all prepared sections, and mean values were determined for each parameter within each group. Ovarian sections were stained using Crossman’s triple staining method to assess corpus luteum angiogenesis, as described by Crossman (1937) [70]. Ovarian follicles at various developmental stages (primordial, primary, secondary, and Graafian) and corpus luteum were counted throughout the ovarian sections, and follicular classification was based on the morphological criteria described by Mazaud et al. (2002) [71]. Additionally, PAS reactivity of the zona pellucida was graded as none (−), weak (+), moderate (++), or severe (+++).

The thickness of the germinative epithelium was measured on H&E-stained ovarian sections [72]. From each paraffin block, five serial ovarian cross-sections of 5 µm thickness were obtained at 30 µm intervals. Germinative epithelium thickness was measured in 10 different representative areas from each ovarian section, and the arithmetic mean of these measurements was calculated for each animal. Sections were examined using a Nikon Eclipse 50i microscope and photographed with a Nikon Digital Sight imaging system. Morphometric measurements were performed using NIS-Elements F Package software, version 3.22 (Nikon Corporation, Tokyo, Japan).

To assess corpus luteum angiogenesis, 5 µm-thick ovarian sections from all experimental groups were stained with H&E staining. Angiogenesis within the corpus luteum was evaluated semi-quantitatively in 10 randomly selected fields at 40× magnification. A histological score was derived from the distribution of stained vessels, 0: no positive vessel in the scanned area (−), +1: 1–2 vessels (+), +2: 3–4 vessels (++), and +3: 5–6 vessels (+++) [73].

For MC counting, 5 µm-thick ovarian sections were stained with 0.5% toluidine blue (pH 0.5; Sigma-Aldrich, CAS 92-31-9) for 10 min, as previously described by Enerbäck, 1966 [74]. MCs were counted in 10 randomly selected fields from ovarian sections in all experimental groups, and the arithmetic mean was calculated. Counting was performed at 40× magnification using a 100-square ocular micrometer (eyepiece graticule), and the results were expressed as the number of MCs per 1 mm^2^ [75]. All histological assessments were conducted by an experienced observer blinded to the experimental groups.

### 2.11. Preparation of Ovarian Tissue for VEGF Immunohistochemistry (IHC) Analysis

Ovarian VEGF expression and localization were assessed by IHC, as previously described [76]. Briefly, five ovarian sections, obtained at 30 µm intervals, were evaluated for VEGF immunoreactivity, and the mean value was calculated for each animal. Following deparaffinization, antigen retrieval was performed on 5 µm ovarian sections. The sections were then incubated overnight at 4 °C with a mouse monoclonal VEGF primary antibody (1:500 dilution; Santa Cruz Biotechnology, Dallas, TX, USA, catalog no. sc-7269). This antibody is reported by the manufacturer to react with human, mouse, and rat VEGF and is recommended for immunohistochemistry on paraffin-embedded sections. Immunoreactivity was determined using a goat anti-polyvalent horseradish peroxidase-conjugated antibody (Patolab, PL-125-HL) and 3,3-diaminobenzidine chromogen (Patolab, PL-125-HD), according to the manufacturer’s instructions. Finally, the sections were counterstained with hematoxylin, mounted with Entellan, and examined under a microscope.

VEGF immunoreactivity was evaluated using a histoscore approach under 20× magnification. Both the intensity and the percentage of VEGF immunostaining were assessed. Immunostaining intensity was graded as negative (0), trace (0.5), light (1), moderate (2), or intense (3). The percentage of VEGF-immunopositive area was scored as follows: 0 (no staining), 0.1 (less than 25% staining), 0.4 (26–50% staining), 0.6 (51–75% staining), and 0.9 (76–100% staining), indicating near homogeneity. To assess both staining intensity and uniformity of VEGF immunostaining simultaneously, a composite histoscore was calculated. The procedure involved multiplying the mean intensity value for each tissue by the mean percentage of stained area in that tissue (histoscore = intensity x area) [77]. For each animal, five serial cross-sections of 5 µm thickness were obtained from each block at 30 µm intervals. Histoscores obtained from the evaluated fields were averaged to calculate a single mean histoscore for each animal. All immunohistochemical evaluations were performed by an experienced observer blinded to the experimental groups.

### 2.12. Statistical Analyses

The normality of the data was assessed using the Q-Q plots and the Shapiro–Wilk test, and the homogeneity of variance was evaluated using the Levene test. For normally distributed data, two-way ANOVA was used to evaluate the main effects of WAS and CoQ10, as well as the WAS × CoQ10 interaction. When a significant interaction effect was detected, Bonferroni-adjusted simple-effects analyses were performed to identify the source of the interaction. To assess the magnitude of the differences, partial eta-squared (ηp^2^) was calculated as an effect size. Partial ηp^2^ values were interpreted using thresholds: ≥ 0.01, ≥ 0.06, and ≥ 0.14, indicating small, medium, and large effects, respectively [78]. Data are presented as mean ± standard error of the mean (SEM) for two-way ANOVA. Statistical analyses were conducted using SPSS version 27.0 (SPSS Inc., Chicago, IL, USA), and graphical figures were prepared with GraphPad Prism version 8.0.1 for Windows (GraphPad Software, San Diego, CA, USA). A *p*-value less than 0.05 was considered significant. For RT-qPCR analysis, fold-change values were calculated using the 2^−ΔΔCt^ method through the Qiagen GeneGlobe Data Analysis Center portal. Because RNA samples were pooled within each group before cDNA synthesis, the RT-qPCR data were presented only as descriptive and exploratory fold-change profiles; no statistical comparisons were performed.

## 3. Results

### 3.1. SIM Test

**Figure 1:**
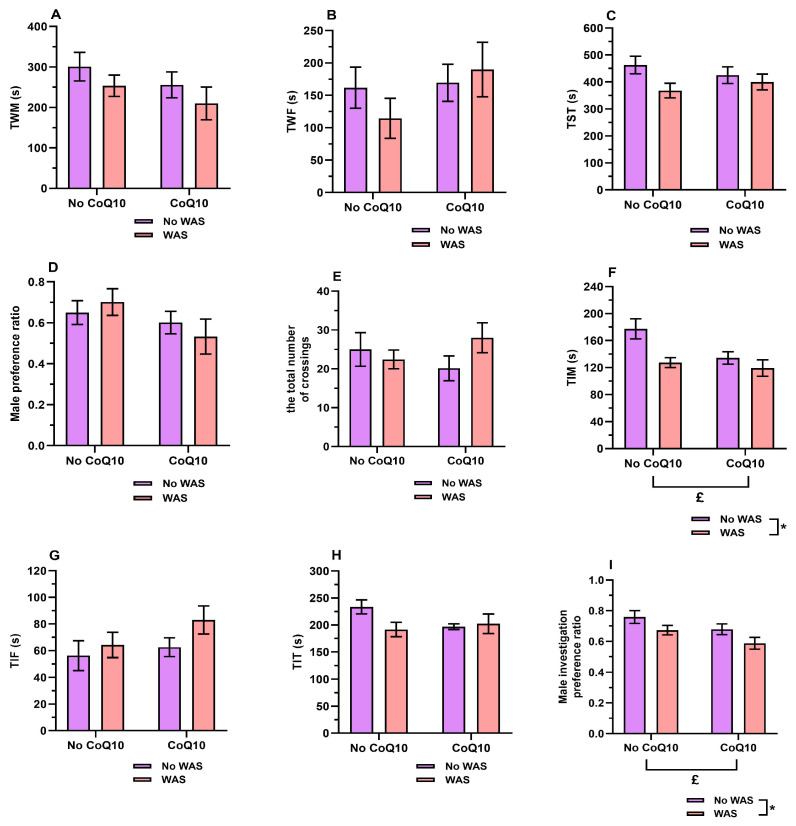
Effects of repeated WAS exposure and/or CoQ10 treatment on sexual incentive motivation parameters and activity: (**A**) TWM, (**B**) TWF, (**C**) TST, (**D**) male preference ratio, and (**E**) the total number of crossings, and active investigation parameters: (**F**) TIM, (**G**) TIF, (**H**) TIT, and (**I**) male investigation preference ratio in adult female rats (Bar graph data are presented as mean ± SEM and were analyzed using two-way ANOVA. * indicates a main effect of WAS, £ indicates a main effect of CoQ10, *p* < 0.05, *n* = 7).

Figure 1A–D illustrates SIM test results. Two-way ANOVA revealed no statistically significant main effects of WAS or CoQ10 and no significant WAS × CoQ10 interaction for TWM, TWF, TST, or male preference ratio (*p* > 0.05 for all effects). Similarly, no statistically significant main effects or interaction were detected for activity (*p* > 0.05, Figure 1E). The two-way ANOVA results showing the main effects of WAS and CoQ10 and their interaction on SIM parameters are presented in Table 2. 

### 3.2. Active Investigation Test

Figure 1F–I presents the findings of the active investigation test. No statistically significant main effects of WAS or CoQ10, or WAS × CoQ10 interaction, were detected for TIF or TIT (Figure 1G,H). The two-way ANOVA results showing the main effects of WAS and CoQ10 and their interaction on behavioral parameters are summarized in Table 2. A two-way ANOVA revealed a significant main effect of WAS on TIM [F(1,24) = 8.343, *p* = 0.008, ηp^2^ = 0.258, Figure 1F], and male investigation preference ratio [F(1,24) = 5.798, *p* = 0.024, ηp^2^ = 0.195, Figure 1I]. Significant main effects of CoQ10 were also observed for TIM [F(1,24) = 5.194, *p* = 0.032, ηp^2^ = 0.178, Figure 1F], and male investigation preference ratio [F(1,24) = 4.876, *p* = 0.037, ηp^2^ = 0.169, Figure 1I]. No significant WAS × CoQ10 interactions were detected for any active investigation parameter (all *p* > 0.05, Table 2). The estimated marginal mean (EMM) TIM was significantly lower in stressed animals (123.36 ± 7.96 s) than in non-stressed animals (155.86 ± 7.96 s, 95% CI: 9.28–55.72, *p* = 0.008), indicating that stress exposure was associated with a significant reduction in TIM regardless of CoQ10 treatment status. Similarly, the EMM male investigation preference ratio was significantly lower in stressed animals (0.630 ± 0.026) than in non-stressed animals (0.719 ± 0.026, 95% CI: 0.013–0.164, *p* = 0.024), indicating that repeated WAS reduced the male investigation preference ratio regardless of CoQ10 treatment status. Regarding the main effects of CoQ10, the EMM TIM was significantly lower in CoQ10-treated animals (126.79 ± 7.96 s) than in animals not receiving CoQ10 (152.43 ± 7.96 s, 95% CI: 2.42–48.87, *p* = 0.032), indicating a significant overall effect of CoQ10 treatment on TIM across stress conditions. Similarly, the EMM male investigation preference ratio was significantly lower in CoQ10-treated animals (0.634 ± 0.026) than in animals not receiving CoQ10 (0.715 ± 0.026, 95% CI: 0.005–0.157, *p* = 0.037), indicating that CoQ10 treatment was associated with a lower male investigation preference ratio regardless of WAS exposure.

### 3.3. Serum Reproductive Hormones and Oxidative Status Parameters

**Figure 2:**
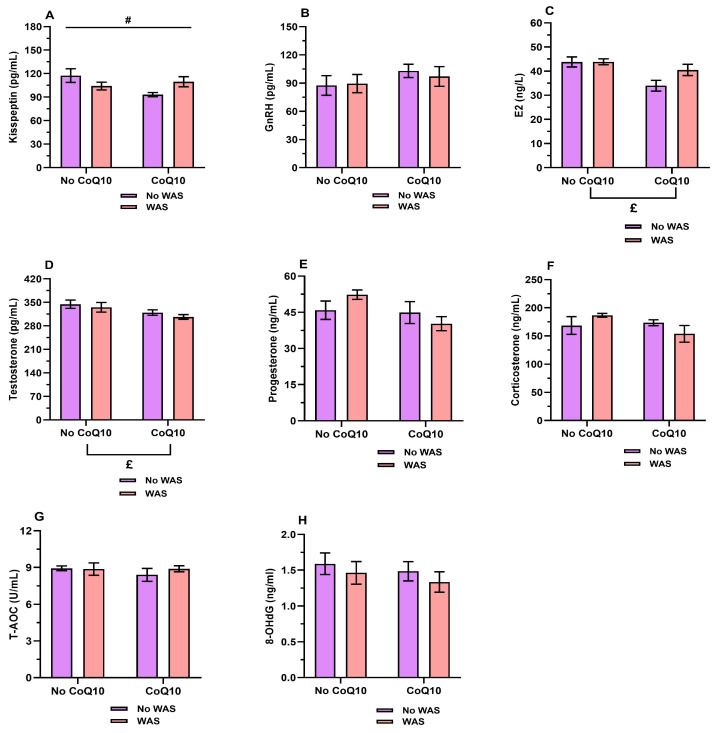
Effects of repeated WAS exposure and/or CoQ10 treatment on serum reproductive hormones, (**A**) kisspeptin, (**B**) GnRH, (**C**) E2, (**D**) testosterone, and (**E**) progesterone, (**F**) corticosterone, (**G**) T-AOC, and (**H**) 8-OHdG levels (Bar graph data are presented as mean ± SEM and were analyzed using two-way ANOVA. £ indicates a main effect of CoQ10 and # indicates a significant WAS × CoQ10 interaction, *p* < 0.05, *n* = 7).

Figure 2A–H illustrates the serum reproductive hormones, corticosterone, T-AOC, and 8-OHdG levels. The two-way ANOVA results showing the main effects of WAS and CoQ10 and their interaction on biochemical parameters are summarized in Table 3. A two-way ANOVA was performed to evaluate the effects of WAS, CoQ10 treatment, and their interaction on serum kisspeptin levels. Neither the main effect of WAS [F(1,24) = 0.064, *p* = 0.802, ηp^2^ = 0.003] nor the main effect of CoQ10 [F(1,24) = 2.391, *p* = 0.135, ηp^2^ = 0.091] was statistically significant. However, a significant WAS × CoQ10 interaction was detected [F(1,24) = 5.944, *p* = 0.023, ηp^2^ = 0.199, Figure 2A], indicating that the effect of CoQ10 on kisspeptin levels differed according to stress condition. Examination of the EMM showed a crossover interaction pattern. In non-stressed animals, CoQ10 treatment significantly decreased kisspeptin levels from 117.55 ± 6.09 pg/mL to 93.28 ± 6.09 pg/mL, as confirmed by Bonferroni-adjusted simple main effects analysis (95% CI: 6.492–42.051, *p* = 0.010). In contrast, in stressed animals, CoQ10 treatment was associated with a numerical increase in kisspeptin levels from 104.24 ± 6.09 pg/mL to 109.67 ± 6.09 pg/mL; however, this difference was not statistically significant (95% CI: −12.348 to 23.211, *p* = 0.534). Similarly, among animals receiving CoQ10, the effect of stress was not statistically significant (109.67 ± 6.09 pg/mL to 93.28 ± 6.09 pg/mL, 95% CI: −1.387 to 34.172, *p* = 0.069). Among animals not receiving CoQ10, kisspeptin levels did not differ significantly between stressed and non-stressed conditions (104.24 ± 6.09 pg/mL vs. 117.55 ± 6.09 pg/mL, 95% CI: −4.470 to 31.090, *p* = 0.135). No significant main effects of WAS were detected for E2, testosterone, progesterone, corticosterone, T-AOC, kisspeptin, GnRH, or 8-OHdG levels (all *p* > 0.05, Table 3). Likewise, no significant main effects of CoQ10 were found for progesterone, corticosterone, T-AOC, kisspeptin, GnRH, or 8-OHdG (all *p* > 0.05). However, significant main effects of CoQ10 were observed for E2 [F(1,24) = 10.665, *p* = 0.003, ηp^2^ = 0.308, Figure 2C] and testosterone levels [F(1,24) = 6.064, *p* = 0.021, ηp^2^ = 0.202, Figure 2D], indicating that CoQ10 treatment significantly influenced both sex hormones regardless of WAS exposure. Examination of the EMM showed that E2 levels were significantly lower in CoQ10-treated animals (37.237 ± 1.436 ng/L) than in animals not receiving CoQ10 (43.867 ± 1.436 ng/L, 95% CI: 2.440–10.820, *p* = 0.003). Similarly, testosterone levels were significantly lower in CoQ10-treated animals (313.207 ± 7.718 pg/mL) than in animals not receiving CoQ10 (340.086 ± 7.718 pg/mL, 95% CI: 4.351–49.406, *p* = 0.021).

### 3.4. RT-qPCR

**Figure 3:**
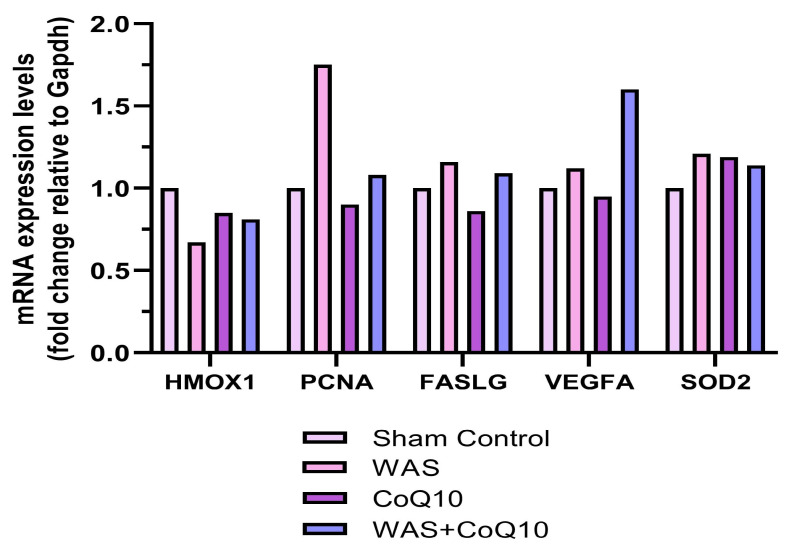
Descriptive ovarian gene expression fold-change profiles of genes associated with oxidative stress response, proliferation, apoptosis, angiogenesis, and antioxidant defense after repeated WAS exposure and/or CoQ10 treatment.

Figure 3 depicts the descriptive RT-qPCR gene expression profiles of ovarian genes associated with oxidative stress response, proliferation, apoptosis, angiogenesis, and antioxidant defense. Because RNA samples from animals within each group were pooled before cDNA synthesis, each group represented a single pooled biological sample. Therefore, the RT-qPCR data were presented only as descriptive and exploratory fold-change values.

### 3.5. Ovary, Uterus, and Adrenal Gland Weights

**Figure 4:**
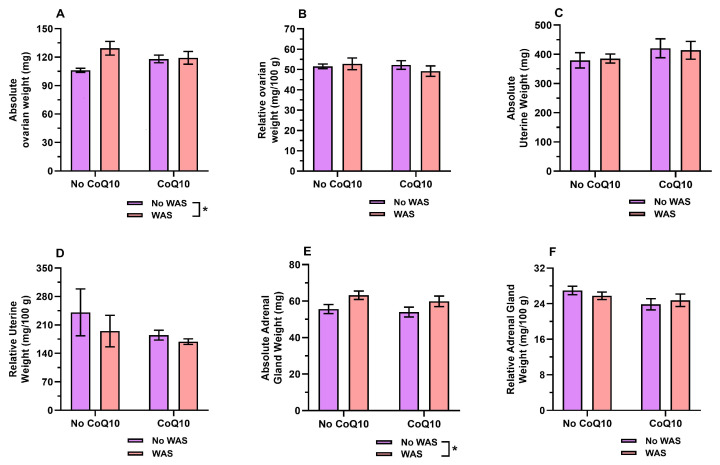
Effects of repeated WAS exposure and/or CoQ10 treatment on absolute and relative weights of the ovaries, uterus, and adrenal glands: (**A**) absolute ovarian weight, (**B**) relative ovarian weight, (**C**) absolute uterine weight, (**D**) relative uterine weight, (**E**) absolute adrenal gland weight, and (**F**) relative adrenal gland weight (Bar graph data are presented as mean ± SEM and were analyzed using two-way ANOVA. * indicates a main effect of WAS *p* < 0.05, *n* = 7).

Figure 4A–F illustrates the effects of repeated WAS protocol and/or CoQ10 treatment on absolute and relative weights of the ovaries, uterus, and adrenal glands. The two-way ANOVA results showing the main effects of WAS and CoQ10 and their interaction on organ weights are summarized in Table 4. A two-way ANOVA revealed significant main effects of WAS on absolute ovarian weight [F(1,24) = 4.966, *p* = 0.035, ηp^2^ = 0.171, Figure 4A] and absolute adrenal gland weight [F(1,24) = 6.729, *p* = 0.016, ηp^2^ = 0.219, Figure 4E]. No statistically significant main effects of CoQ10 treatment were observed for absolute or relative ovarian, uterine, or adrenal gland weights, and no significant WAS × CoQ10 interactions were detected for any absolute or relative organ weights (all *p* > 0.05, Table 4). Examination of the EMM showed that ovarian weight was significantly higher in WAS-exposed animals (124.350 ± 3.878 mg) than in non-stressed animals (112.129 ± 3.878 mg, 95% CI: 0.903–23.540, *p* = 0.035). Similarly, adrenal gland weight was significantly higher in WAS-exposed animals (61.536 ± 1.830 mg) than in non-stressed animals (54.821 ± 1.830 mg, 95% CI: 1.372–12.056, *p* = 0.016). In contrast, relative ovarian, uterine, and adrenal gland weights did not differ significantly by WAS exposure, CoQ10 treatment, or their interaction (all *p* > 0.05; Table 4).

### 3.6. Histological Effects

**Figure 5:**
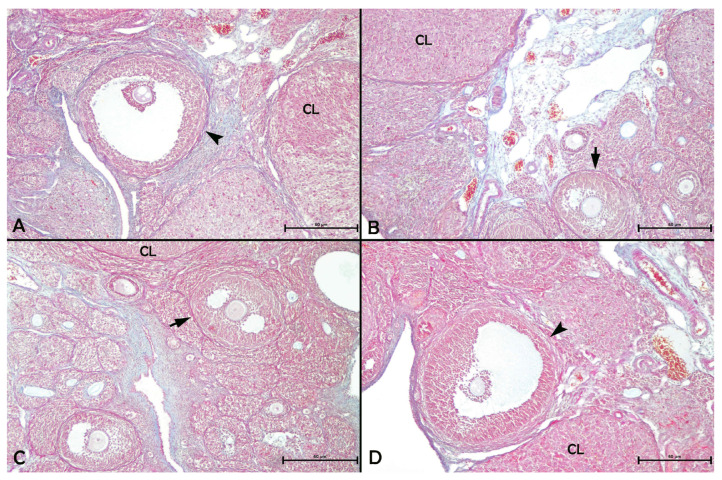
Histological micrographs of the ovarian histology following repeated WAS exposure and/or CoQ10 treatment. (**A**) Sham control, (**B**) WAS, (**C**) CoQ10, and (**D**) WAS + CoQ10. Crossman’s triple staining, CL: Corpus luteum, Arrowhead: Graafian follicle, Arrow: Secondary follicle. Scale bar = 50 µm; ×10 magnification.

**Figure 6:**
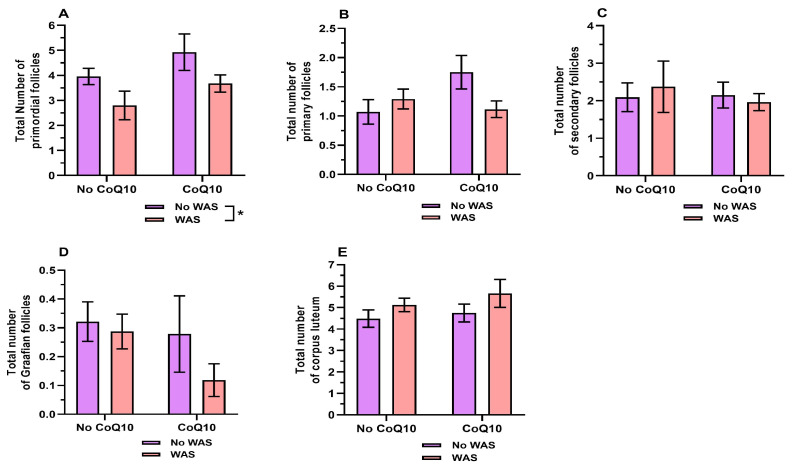
Effects of repeated WAS exposure and/or CoQ10 treatment on (**A**) the total number of primordial, (**B**) primary, (**C**) secondary, and (**D**) Graafian follicles, and (**E**) the total number of corpus luteum (Bar graph data are presented as mean ± SEM and were analyzed using two-way ANOVA. * indicates a main effect of WAS, *n* = 7).

**Figure 7:**
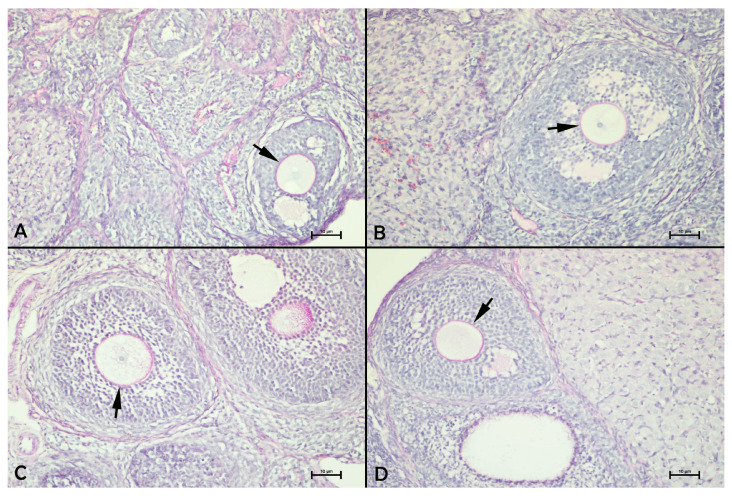
Representative PAS-stained ovarian sections showing zona pellucida reactivity following repeated WAS exposure and/or CoQ10 treatment. (**A**) Sham control, (**B**) WAS, (**C**) CoQ10, and (**D**) WAS + CoQ10: periodic acid Schiff staining, Scale bar = 10 µm; 20× magnification. The arrow indicates the zona pellucida.

Figure 5A–D illustrates representative micrographs of follicles at different developmental stages. The two-way ANOVA results showing the main effects of WAS and CoQ10 and their interaction on the total numbers of primordial, primary, secondary, and Graafian follicles, as well as the number of corpus lueteum, are summarized in Table 5. A two-way ANOVA revealed a significant main effect of WAS on the total number of primordial follicles [F(1,24) = 5.327, *p* = 0.030, ηp^2^ = 0.182, Figure 6A], indicating that stress exposure significantly influenced the number of primordial follicles. Examination of the EMM showed that the total number of primordial follicles was significantly lower in WAS-exposed animals (3.239 ± 0.369) than in non-stressed animals (4.443 ± 0.369, 95% CI: 0.127–2.281, *p* = 0.030). No statistically significant main effects of CoQ10 treatment or WAS × CoQ10 interactions were observed for any follicular count or the total number of corpus luteum (all *p* > 0.05). Regarding PAS reactivity of the zona pellucida, the zona pellucida exhibited severe (+++) staining in the sham control group, moderate (++) staining in both the WAS and CoQ10 groups, and severe (+++) staining in the WAS + CoQ10 group (Figure 7A–D).

**Figure 8:**
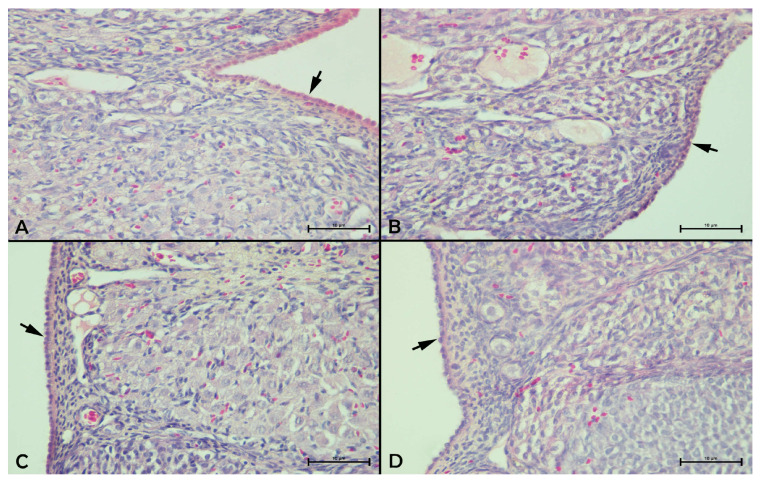
Representative ovarian sections illustrating germinative epithelium morphology following repeated WAS exposure and/or CoQ10 treatment. (**A**) Sham control, (**B**) WAS, (**C**) CoQ10, and (**D**) WAS + CoQ10: Hematoxylin and eosin (H&E), Scale bar = 10 µm; 40× magnification. The arrow indicates the germinative epithelium.

**Figure 9:**
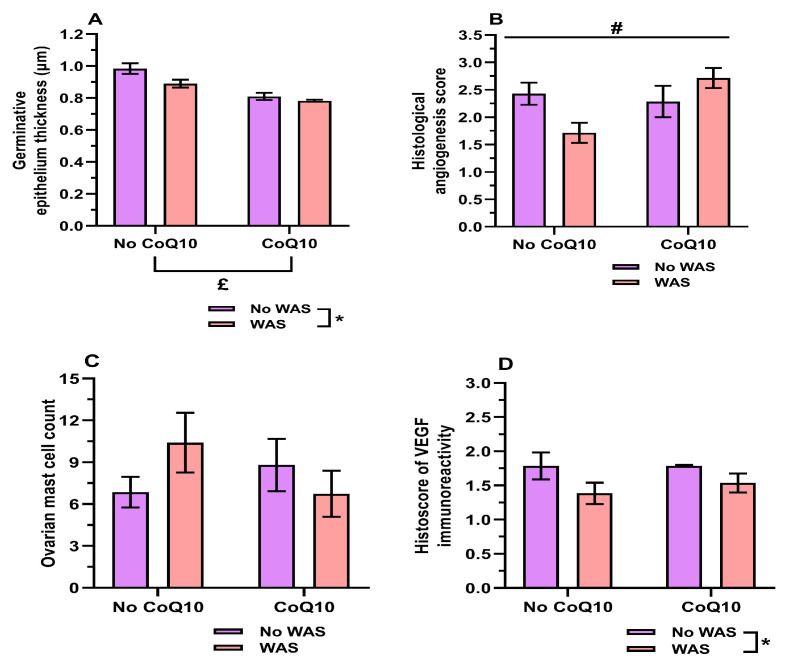
Effects of repeated WAS exposure and/or CoQ10 treatment on (**A**) germinative epithelium thickness, (**B**) histological angiogenesis score, (**C**) ovarian mast cell counts, and (**D**) histoscore of VEGF immunoreactivity (Bar graph data are presented as mean ± SEM and were analyzed using two-way ANOVA. * indicates a main effect of WAS, £ indicates a main effect of CoQ10 and # indicates a significant WAS × CoQ10 interaction, *p* < 0.05, *n* = 7).

Figure 8A–D shows representative images of germinative epithelium thickness across the experimental groups. The two-way ANOVA results showing the main effects of WAS and CoQ10, and their interaction, on germinative epithelium thickness are summarized in Table 6. A two-way ANOVA revealed significant main effects of WAS [F(1,24) = 6.552, *p* = 0.017, ηp^2^ = 0.214, Figure 9A] and CoQ10 treatment [F(1,24) = 35.196, *p* < 0.001, ηp^2^ = 0.595, Figure 9A] on germinative epithelium thickness, whereas no statistically significant WAS × CoQ10 interaction was detected. Examination of the EMM showed that germinative epithelium thickness was significantly lower in WAS-exposed animals (0.836 ± 0.017 µm) than in non-stressed animals (0.897 ± 0.017 µm, 95% CI: 0.012–0.110, *p* = 0.017). In addition, germinative epithelium thickness was significantly lower in CoQ10-treated animals (0.796 ± 0.017 µm) than in animals not receiving CoQ10 (0.937 ± 0.017 µm, 95% CI: 0.092–0.190, *p* < 0.001), indicating a substantial overall effect of CoQ10 treatment on ovarian epithelial morphology. 

**Figure 10:**
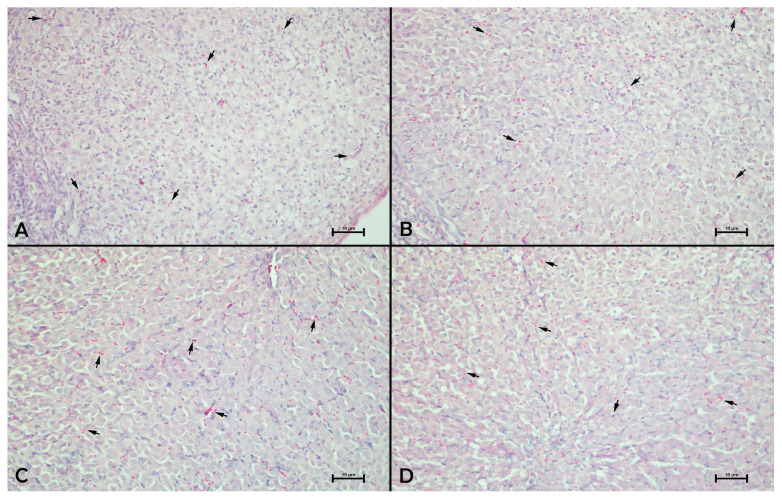
Representative ovarian sections depicting angiogenesis within the corpus luteum following repeated WAS exposure and/or CoQ10 treatment. (**A**) Sham control, (**B**) WAS, (**C**) CoQ10, and (**D**) WAS + CoQ10: Hematoxylin and eosin (H&E), Scale bar = 10 µm; 20× magnification. The arrow indicates angiogenesis in the corpus luteum.

Figure 10A–D illustrates corpus luteum angiogenesis in the experimental groups. The two-way ANOVA results showing the main effects of WAS and CoQ10 and their interaction on corpus luteum angiogenesis are summarized in Table 6. For corpus luteum angiogenesis, a significant WAS × CoQ10 interaction was observed [F(1,24) = 6.857, *p* = 0.015, ηp^2^ = 0.222, Figure 9B], whereas neither the main effect of WAS [F(1,24) = 0.429, *p* = 0.519, ηp^2^ = 0.018] nor the main effect of CoQ10 treatment [F(1,24) = 3.857, *p* = 0.061, ηp^2^ = 0.138] reached statistical significance. Bonferroni-adjusted simple main effects analysis demonstrated a crossover interaction pattern. Corpus luteum angiogenesis did not differ significantly between CoQ10-treated and untreated animals under non-stressed conditions (2.286 ± 0.218 vs. 2.429 ± 0.218, 95% CI: −0.494 to 0.780, *p* = 0.648). 

**Figure 11:**
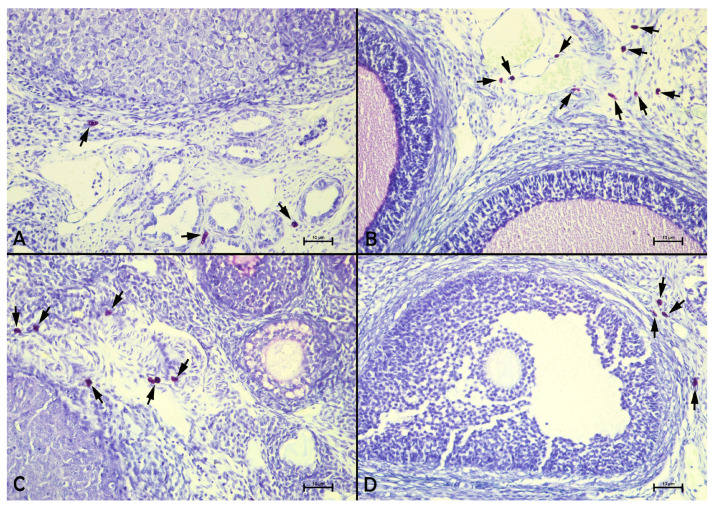
Representative ovarian sections showing ovarian mast cell staining with toluidine blue following repeated WAS exposure and/or CoQ10 treatment. (**A**) Sham control, (**B**) WAS, (**C**) CoQ10, and (**D**) WAS + CoQ10: Scale bar = 10 µm; 20× magnification. The arrow indicates a mast cell.

**Figure 12:**
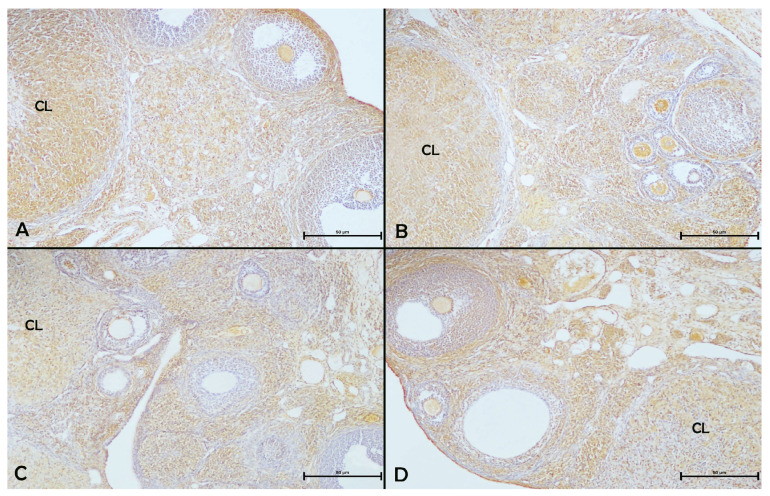
Representative ovarian sections illustrating VEGF immunoreactivity following repeated WAS exposure and/or CoQ10 treatment. (**A**) Sham control, (**B**) WAS, (**C**) CoQ10, and (**D**) WAS + CoQ10. CL: Corpus luteum. Scale bar = 50 µm; 10× magnification.

In contrast, under stress conditions, CoQ10 treatment significantly increased corpus luteum angiogenesis (1.714 ± 0.218 to 2.714 ± 0.218, 95% CI: 0.363–1.637, *p* = 0.003). Similarly, WAS exposure significantly reduced corpus luteum angiogenesis in animals not receiving CoQ10 treatment (2.429 ± 0.218 vs. 1.714 ± 0.218, 95% CI: 0.077–1.351, *p* = 0.030), whereas no significant effect of WAS was observed among CoQ10-treated animals (2.286 ± 0.218 vs. 2.714 ± 0.218, 95% CI: −0.208 to 1.066, *p* = 0.178). Figure 11A–D presents representative ovarian sections stained for MCs. No statistically significant main effects of WAS or CoQ10 treatment, or WAS × CoQ10 interaction, were observed for mast cell count (all *p* > 0.05, Figure 9C, Table 6). Figure 12A–D depicts VEGF immunoreactivity in ovarian sections. 

The two-way ANOVA results showing the main effects of WAS and CoQ10 and their interaction on VEGF histoscore are summarized in Table 6. A significant main effect of WAS was detected for VEGF histoscore [F(1,24) = 5.115, *p* = 0.033, ηp^2^ = 0.176, Figure 9D], whereas no significant main effect of CoQ10 treatment or WAS × CoQ10 interaction was observed. The EMM VEGF histoscore was significantly lower in WAS-exposed animals (1.461 ± 0.102) than in non-stressed animals (1.786 ± 0.102, 95% CI: 0.028–0.622, *p* = 0.033).

## 4. Discussion

This study evaluated the effects of repeated WAS and/or CoQ10 treatment on female reproductive function by assessing behavioral, endocrine, oxidative, histological, angiogenic, and immune-related outcomes. Overall, WAS mainly reduced active investigation behaviors, primordial follicle number, germinative epithelium thickness, corpus luteum angiogenesis, and VEGF immunoreactivity, while increasing ovarian and adrenal weights. CoQ10 mainly reduced E2, testosterone, and germinative epithelium thickness but increased corpus luteum angiogenesis under stress conditions.

### 4.1. Reproductive Behavioral, Endocrine, and Molecular Outcomes

Sexual incentive motivation in rats is assessed using the SIM test protocol. It is described as the time spent in a zone adjacent to the inaccessible sexual stimulus relative to the time spent in another zone adjacent to the inaccessible social stimulus [66]. In this test, receptive female rats, either in the naturally proestrous phase or those that received E2 and progesterone, exhibit a higher preference for a sexually active male rat than rats in the diestrous phase or OVX [79]. Despite the test’s established utility, to date, no studies have investigated the effects of WAS and/or CoQ10 treatment on female sexual incentive motivation in rats in combination with the neuroendocrine profile. No statistically significant main effects of WAS or CoQ10, or WAS × CoQ10 interaction, were detected for TWM, TWF, TST, or the male preference ratio under the present experimental conditions. No statistically significant change in locomotor activity was also detected, indicating that the SIM parameters assessed here were not significantly altered by the present WAS protocol and treatment schedule. Studies examining the effects of stress and/CoQ10 on sexual motivation in female rats are extremely limited, and previous studies have primarily focused on the effects of chronic stress on the consummatory aspect of female sexual behavior [20,31,80].

In the most relevant previous study, the effects of acute mild restraint stress on female sexual motivation were examined in hormonally primed [EB or EB + progesterone] OVX rats. Consistent with our results, acute mild restraint stress did not affect either sexual or social motivation [81]. Similarly, in the same study, the number of crossings at the apparatus center did not change. This comparison should nevertheless be interpreted with caution, as the previous study was conducted in OVX-hormonally primed rats [81], whereas our study used naturally cycling intact females. Even though we acknowledge this methodological difference, the two models are not entirely divergent, as female sexual behavior can be examined in both intact, naturally cycling, and OVX females. In intact models, vaginal smears are usually collected daily to evaluate estrous cyclicity, and behavioral estrus is verified on the test day by exposing the female to a sexually experienced male rat.

However, in OVX models, hormonal priming with EB and progesterone at doses known to induce behavioral estrus is conducted to induce artificial estrus after ovariectomy [82]. A study comparing both models with respect to sexual motivation reported a similar overall pattern of partner preference in proestrous rats to that observed in estrogen- and progesterone-primed OVX rats [83]. Collectively, these observations demonstrate that, despite methodological differences between the two models, the unchanged SIM parameters observed in our study align with the limited available literature. However, this finding should not be interpreted as evidence that stress generally does not affect female sexual incentive motivation; rather, it indicates that sexual incentive motivation was not significantly altered under the present WAS protocol and experimental conditions. In contrast, the active investigation test revealed significant main effects of both WAS and CoQ10 on TIM and the male investigation preference ratio, with lower values observed in stressed animals and in CoQ10-treated animals. Since no significant WAS × CoQ10 interaction was detected, these findings suggest that repeated WAS and CoQ10 treatment independently reduced active investigation behaviors under the present experimental conditions.

From a translational perspective, clinical studies generally indicate that psychological stress is associated with poorer female sexual function, including reduced sexual desire, arousal, satisfaction, and sexual activity [25,84,85,86]. In the present study, repeated WAS did not significantly change sexual incentive motivation parameters, whereas male-directed active investigation behaviors were reduced. This suggests that WAS was not associated with a broad pattern of statistically detectable impairment in sexual incentive motivation under the present experimental conditions, but may have affected a more limited behavioral component related to direct investigation of the sexual stimulus. Therefore, the present findings only partially parallel clinical observations in women under stress. This difference should be interpreted cautiously, because sexual desire in women includes biological, psychological, social, and contextual dimensions that female rat behavioral tests cannot fully represent [28]. Thus, the present behavioral findings may provide limited translational insight into the investigatory or approach-related aspect of female sexual motivation. However, they should not be considered to fully model stress-associated reductions in women’s sexual desire.

Neuroendocrine and antioxidant profile examination demonstrated that no statistically significant WAS-related changes were detected in serum E2, progesterone, testosterone, GnRH, kisspeptin, corticosterone, 8-OHdG, or T-AOC levels. A review of the available literature suggests that studies exploring the effects of WAS on the female reproductive system and neuroendocrine hormones remain scarce. Previous studies using chronic stress paradigms in female rodents have reported heterogeneous endocrine responses. Combined restraint and forced swimming stress did not alter E2 or testosterone levels after 4 weeks [40], and chronic restraint stress similarly did not change E2 levels after 28 days [87]. In contrast, cold stress decreased E2, progesterone, and testosterone after 7, 14, and 21 days [23]. Heat stress produced a more hormone-specific pattern, reducing E2 after 90 days while leaving testosterone, progesterone, and GnRH unchanged [88]. These findings indicate that stress-induced endocrine responses in female rodents are not uniform across models, exposure durations, or hormones. Therefore, the absence of significant hormonal changes after repeated WAS in the present study should be interpreted cautiously. It may reflect a model- and hormone-specific response under the present experimental conditions; however, alternative explanations such as terminal sampling time, adaptation after repeated exposure, or stress intensity insufficient to alter circulating hormone levels cannot be excluded. Although the sample size was supported by power analysis, the possibility of smaller endocrine effects remaining undetected should also be considered.

Regarding corticosterone, previous WAS studies typically show that this stress model activates the HPA axis and elevates circulating corticosterone levels in rodents. Peng et al. (2012) reported that 10 days of WAS increased corticosterone levels in female rats, although the exact timing of blood collection after the final stress session was not specified [89]. Similarly, in female mice, West et al. (2022a,b) demonstrated elevated corticosterone levels 24 h after the final session of a 10-day WAS protocol [90,91]. In the present study, however, repeated WAS did not significantly alter serum corticosterone levels. This discrepancy may be related to protocol-dependent factors, including species, stress exposure conditions, and, in particular, the timing of blood collection after the final WAS session. Therefore, unchanged corticosterone levels should not be interpreted as evidence that WAS failed to induce a stress-related response. Indeed, repeated WAS significantly increased absolute adrenal gland weight, although relative adrenal weight remained unchanged. Adrenal hypertrophy is considered a typical and sensitive marker of HPA axis activation in response to stress [92,93,94]. This finding supports the presence of stress-related HPA axis activation despite the absence of a detectable corticosterone change at the terminal sampling point.

Regarding the effects of CoQ10 on the neuroendocrine profile, CoQ10 significantly reduced serum E2 and testosterone levels regardless of WAS exposure, whereas progesterone, GnRH, corticosterone, 8-OHdG, and T-AOC levels did not change. In addition, CoQ10 significantly reduced kisspeptin-1 levels under non-stressed conditions, but this effect was not observed under stressed conditions. These findings suggest that CoQ10 did not produce a generalized suppression of the HPO axis but may exert hormone-specific endocrine-modulating effects under the present experimental conditions. Available literature demonstrates that the endocrine effects of CoQ10 are not uniform, and previously reported hormonal changes after CoQ10 treatment may differ depending on pathological settings, such as PCOS or experimental ovarian hyperstimulation syndrome (OHSS), rather than after CoQ10 administration alone in otherwise healthy females. In women with PCOS, CoQ10 has reduced testosterone levels [95,96]. Similarly, in female rat models of experimental OHSS or obesity, CoQ10 treatment has been associated with reduced serum E2 levels, while progesterone did not change. However, CoQ10 treatment alone did not alter serum E2 or progesterone levels [97,98]. Consistent with these results, a study in female mice similarly found that CoQ10 did not alter E2 levels [99]. Therefore, the reduction in E2 and kisspeptin-1 observed under non-stressed conditions in the present study may indicate that CoQ10 can modulate endocrine signaling even in otherwise healthy animals. This finding may represent a physiological endocrine modulation or a potentially unfavorable hormonal effect under non-stressed conditions, rather than a clearly beneficial effect. Nevertheless, because progesterone, GnRH, and corticosterone remained unchanged, and the kisspeptin-1 reduction was not observed under stressed conditions, the present data do not support a generalized inhibitory effect of CoQ10 on the HPO axis. Further studies are required to clarify whether these effects depend on dose, treatment duration, estrous-cycle stage, or baseline endocrine status.

### 4.2. Ovarian Histological Parameters

Our study showed that repeated WAS significantly reduced the total number of primordial follicles. In contrast, no statistically significant changes were detected in primary, secondary, or Graafian follicle counts or the number of corpus luteum. CoQ10 treatment did not significantly affect any follicular or corpus luteum count, and no statistically significant WAS × CoQ10 interaction was observed. These findings suggest that repeated WAS may preferentially affect the primordial follicle pool under the present experimental conditions. Findings from previous studies indicate that chronic stress does not affect all stages of follicular development uniformly, but rather produces model- and stage-dependent effects.

For example, in female rats exposed to CUMS, Sun et al. (2017) reported no change in primordial or secondary follicle counts, whereas primary follicles and corpora lutea decreased [100]. In female mice, a different stage-specific pattern was observed, in which chronic restraint stress decreased primordial and secondary follicles and corpora lutea while simultaneously increasing primary follicle counts [101]. Other mouse studies provide additional support for this stage-specific variability. Xiang et al. (2022) revealed that CUMS did not change primordial or primary follicle numbers but decreased secondary follicles [102]. Conversely, Kim and You (2022) reported that high-housing-density-induced chronic stress decreased primordial and primary follicles without impacting secondary follicles [103]. Taken together, these results reveal inconsistent ovarian response to chronic stress across models and follicular stages. In the present study, repeated WAS reduced the primordial follicle number but did not produce a generalized decrease in later follicular stages or the number of corpus luteum. Therefore, the follicular effect of WAS appears to be stage-specific under the present experimental conditions.

Consistent with our results, previous studies in female mice and rats did not show changes in primordial, primary, secondary, or Graafian follicle numbers or corpus luteum counts following CoQ10 administration across several models [61,97,98,104]. Accordingly, CoQ10 treatment was not associated with statistically detectable changes in follicular counts or the number of corpus luteum in the present study. This suggests that the ovarian effects of CoQ10 may be reflected more in endocrine or histomorphological changes than in measurable alterations in follicle reserve or the number of corpus luteum.

For the PAS reactivity of the zona pellucida, severe (+++) staining in the sham control group and moderate (++) staining in both the WAS and CoQ10 groups were observed. In contrast, the WAS + CoQ10 group exhibited severe (+++) staining compared with the sham control group. The mammalian oocyte zona pellucida is a porous extracellular matrix composed of three glycoproteins that together form its characteristic fibrogranular architecture [105]. This extracellular matrix, which surrounds the mammalian oocyte, is pivotal in gamete recognition and binding, fertilization, and the early embryonic development [106]. Consistent with this glycoprotein-rich composition, PAS staining is highly used to determine polysaccharide-containing tissue components, including glycogen, glycoproteins, glycolipids, and mucins [107]. To our current knowledge, no previous study has examined the effects of WAS-induced psychogenic stress or CoQ10 treatment on zona pellucida PAS reactivity. Several studies demonstrate that faint PAS reactions in the zona pellucida have been regarded as indicative of reduced or depleted carbohydrate content within the oocyte and its surrounding zona pellucida [108,109]. Therefore, the reduced PAS reactivity of the zona pellucida in our study indicates a weakening of carbohydrate/glycoprotein-rich components of the oocyte–zona pellucida matrix. Hence, the moderate PAS reactivity observed in both the WAS and CoQ10 groups demonstrates that each treatment may individually modify the carbohydrate structure of the zona pellucida. Nevertheless, the severe (+++) PAS staining in the WAS + CoQ10 group suggests that their combination did not exert an additive adverse effect. Instead, CoQ10 may have interacted with the stress response in a context-dependent manner, retaining zona pellucida PAS reactivity under WAS conditions.

In histomorphological assessments, two-way ANOVA showed significant main effects of both WAS and CoQ10 on germinative epithelium thickness, with lower values observed in WAS-exposed animals and in CoQ10-treated animals. No significant WAS × CoQ10 interaction was detected. Ovarian germinative epithelium, also termed ovarian surface epithelium (OSE), denotes a highly dynamic ovarian structure. It allows for ovarian follicle formation during development, undergoes repeated cyclic repair following ovulatory rupture throughout the reproductive period, and eventually exhibits functional and structural decline with reproductive aging. Depending on its location and the stage of the ovarian cycle, OSE consists of a simple layer of squamous-to-cuboidal cells [110]. In the present study, germinative epithelium thickness was reduced in relation to both WAS exposure and CoQ10 treatment. Together with the reduction in primordial follicle number and VEGF immunoreactivity after WAS, decreased germinative epithelium thickness may reflect a selective ovarian structural response. However, the absence of broad changes in later follicular stages, in the number of corpora lutea, and in mast cell counts suggests that this finding should not be interpreted as evidence of generalized ovarian damage.

For other histological parameters, corpus luteum angiogenesis showed a significant WAS × CoQ10 interaction. CoQ10 increased corpus luteum angiogenesis under stress conditions, whereas WAS reduced this parameter in animals not receiving CoQ10. These findings suggest that CoQ10 may modulate luteal angiogenic morphology under WAS conditions. In contrast, ovarian MC counts remained unchanged across the experimental conditions. However, VEGF immunoreactivity was significantly reduced by WAS, indicating that repeated WAS may affect selected angiogenesis-related components of the ovarian microenvironment.

Corpus luteum angiogenesis is functionally important because the corpus luteum is characterized by intense angiogenic activity, and ovarian vascular remodeling supports oxygen, nutrient, and hormone precursor delivery to the corpus luteum [111,112]. VEGF is one of the major regulators of ovarian angiogenesis and is very important for follicular development and corpus luteum formation [38]. Previous studies suggest that stress can affect the vascular component of the female reproductive system; restraint stress has been associated with reduced micro-vessel density and VEGF expression, while cold stress has been shown to impair ovarian and uterine microcirculation [23,113]. In addition, reduced serum VEGF levels have been reported in a chronic stress-induced premature ovarian failure model [114]. Together, these findings indicate that chronic stress may reduce VEGF-related angiogenic activity and angiogenesis in the female reproductive system. In the present study, CoQ10 increased corpus luteum angiogenesis under WAS conditions, whereas WAS reduced this parameter in animals not receiving CoQ10. Together with the WAS-related reduction in VEGF immunoreactivity, these findings suggest that repeated WAS may affect selected vascular components of the ovarian microenvironment. Although direct evidence regarding CoQ10-mediated regulation of luteal angiogenesis under psychogenic stress is limited, previous experimental evidence indicates that CoQ10 can regulate ovarian angiogenesis-related signaling in pathological ovarian conditions such as ovarian hyperstimulation syndrome by reducing ovarian VEGF expression [97]. Therefore, the present finding should be interpreted as a possible context-dependent modulatory effect of CoQ10 on corpus luteum angiogenesis under stress conditions, rather than as conclusive evidence of a direct vascular restorative effect.

### 4.3. Limitations of the Study

Several limitations should be acknowledged. First, the relatively short duration of the stress protocol (10 days) and the use of a single terminal time point for hormone and tissue collection may have limited the detection of time-dependent or transient stress responses, including corticosterone changes. Second, oocyte quality and fertility outcomes were not assessed; therefore, the functional relevance of the observed ovarian alterations requires further investigation. Third, a positive control stress group, such as restraint stress, was not included, and additional HPA axis or behavioral validation measures were not assessed, which limits direct comparison with a well-established stress paradigm and should be considered when interpreting the WAS-related findings. Furthermore, since the WAS-only group did not undergo daily oral gavage, unlike the other experimental groups, this difference in handling procedures should be considered as a potential confounding factor when interpreting comparisons involving this group. Moreover, although VEGF IHC was performed using a previously optimized protocol, the absence of separate positive and negative staining controls in the current experimental series should be considered when interpreting VEGF immunoreactivity findings. Although the sample size was supported by power analysis, the possibility of Type II error for some outcomes cannot be excluded. In addition, a global Benjamini–Hochberg FDR correction was not applied across all endpoint families because the study was exploratory and included biologically distinct outcome domains; applying a single correction across all outcomes may therefore be overly conservative and increase the risk of Type II error. Nevertheless, residual Type I error inflation cannot be excluded, and significant findings should be interpreted with caution. Finally, ovarian gene expression analyses were performed using pooled samples, which may have reduced the ability to detect individual biological variability.

## 5. Conclusions

In conclusion, under the present experimental conditions, repeated WAS was not associated with a generalized reproductive dysfunction in adult female rats but was accompanied by reduced active investigation behaviors and selected ovarian structural and vascular alterations. These changes occurred without broad alterations in systemic oxidative status. CoQ10 did not show a broadly protective or uniformly beneficial effect against WAS-related reproductive alterations in this model. Rather, it lowered E2 and testosterone levels, decreased kisspeptin-1 under non-stressed conditions, and reduced active investigation behaviors, while increasing corpus luteum angiogenesis under WAS conditions. Overall, these findings suggest that repeated WAS did not cause broad reproductive impairment in this model but did produce limited behavioral and ovarian changes. CoQ10 has context-dependent effects, and its independent endocrine actions should be further investigated before any translational or clinical recommendations can be made.

## Figures and Tables

**Table 1:** Primary list of oxidative stress, antioxidant defense, proliferation, apoptosis, and angiogenesis-related genes used for RT-qPCR.

Gen Name	Primer Name	Primer Sequence (5′—3′)	NCBI Gene ID	NCBI Access Number	Size (bp)
Heme oxygenase 1	Rno-HMOX1-forward	GTAAATGCAGTGTTGGCCCC	24451	NM_012580.2	178
	Rno-HMOX1-reverse	ATGTGCCAGGCATCTCCTTC			
Proliferating cell nuclear antigen	Rno-PCNA-forward	AGGACGGGGTGAAGTTTTCT	25737	NM_022381.3	173
	Rno-PCNA-reverse	CAGTGGAGTGGCTTTTGTGA			
Fas ligand	Rno-FASL-forward	TGGTGGCTCTGGTTGGAATG	25385	NM_012908.1	77
	Rno-FASL-reverse	CTCACGGAGTTCTGCCAGTT			
Vascular endothelial growth factor A	Rno-VEGFA-forward	TGGGAGGGAAGCTCTTAGGT	83785	NM_001110335.1	155
	Rno-VEGFA-reverse	GGAGCACTTGCTCTTCTGGA			
Superoxide dismutase 2	Rno-SOD2-forward	AGGAGCAAGGTCGCTTACAG	24787	NM_017051.2	98
	Rno-SOD2-reverse	CTCCCACACATCAATCCCCA			
Glyceraldehyde-3-phosphate dehydrogenase *	Rno-GAPDH-forward	AGACAGCCGCATCTTCTTGT	24383	NM_017008.4	207
	Rno-GAPDH-reverse	CTTGCCGTGGGTAGAGTCAT			

* Used as a reference gene.

**Table 2:** Two-way ANOVA results showing the main effects of WAS and CoQ10 and their interaction on behavioral parameters.

Parameters	WAS F(1,24)	*p*	ηp^2^	CoQ10 F(1,24)	*p*	ηp^2^	WAS × CoQ10 F(1,24)	*p*	ηp^2^
TWM	1.874	0.184	0.072	1.706	0.204	0.066	0.000	0.985	0.000
TWF	0.158	0.694	0.007	1.506	0.232	0.059	1.006	0.326	0.040
TST	4.002	0.057	0.143	0.010	0.923	0.000	1.326	0.261	0.052
Male preference ratio	0.028	0.867	0.001	2.663	0.116	0.100	0.809	0.377	0.033
Activity	0.567	0.459	0.023	0.010	0.920	0.000	2.208	0.150	0.084
TIM	8.343	**0.008**	**0.258**	5.194	**0.032**	**0.178**	2.419	0.133	0.092
TIF	2.156	0.155	0.082	1.667	0.209	0.065	0.412	0.527	0.017
TIT	1.902	0.181	0.073	0.983	0.331	0.039	3.199	0.086	0.118
Male investigation preference ratio	5.798	**0.024**	**0.195**	4.876	**0.037**	**0.169**	0.013	0.909	0.001

Data are presented as F values, *p* values, and partial eta squared (ηp^2^) obtained from two-way ANOVA. Bold values indicate statistical significance at *p* < 0.05.

**Table 3:** Two-way ANOVA results showing the main effects of WAS and CoQ10 and their interaction on biochemical parameters.

Parameters	WAS F(1,24)	*p*	ηp^2^	CoQ10 F(1,24)	*p*	ηp^2^	WAS × CoQ10 F(1,24)	*p*	ηp^2^
E2	2.640	0.117	0.099	**10.665**	**0.003**	**0.308**	2.554	0.123	0.096
Testosterone	1.038	0.318	0.041	**6.064**	**0.021**	**0.202**	0.031	0.862	0.001
Progesterone	0.073	0.790	0.003	3.570	0.071	0.129	2.603	0.120	0.098
Corticosterone	0.004	0.949	0.000	1.528	0.228	0.060	2.847	0.105	0.106
T-AOC	0.301	0.588	0.012	0.411	0.528	0.017	0.477	0.496	0.020
Kisspeptin	0.064	0.802	0.003	2.391	0.135	0.091	**5.944**	**0.023**	**0.199**
GnRH	0.045	0.833	0.002	1.459	0.239	0.057	0.173	0.681	0.007
8-OHdG	0.900	0.352	0.036	0.617	0.440	0.025	0.006	0.938	0.000

Data are presented as F values, *p* values, and partial eta squared (ηp^2^) obtained from two-way ANOVA. Bold values indicate statistical significance at *p* < 0.05.

**Table 4:** Two-way ANOVA results showing the main effects of WAS and CoQ10 and their interaction on absolute and relative organ weights.

Parameters	WAS F(1,24)	*p*	ηp^2^	CoQ10 F(1,24)	*p*	ηp^2^	WAS × CoQ10 F(1,24)	*p*	ηp^2^
Abs. ovarian w.	**4.966**	**0.035**	0.171	0.027	0.870	0.001	4.070	0.055	0.145
R. ovary w.	0.146	0.706	0.006	0.412	0.527	0.017	0.869	0.361	0.035
Abs. uterin w.	0.000	0.988	0.000	1.675	0.208	0.065	0.060	0.808	0.003
R. uterin w.	0.766	0.390	0.031	1.345	0.258	0.053	0.178	0.677	0.007
Abs. adrenal gland w.	**6.729**	**0.016**	0.219	0.922	0.346	0.037	0.106	0.748	0.004
R. adrenal gland w.	0.016	0.900	0.001	3.229	0.085	0.119	0.852	0.365	0.034

Data are presented as F values, *p* values, and partial eta squared (ηp^2^) obtained from two-way ANOVA. Bold values indicate statistical significance at *p* < 0.05. Abbreviations: Abs., absolute; R., relative; w., weight.

**Table 5:** Two-way ANOVA results showing the main effects of WAS and CoQ10 and their interaction on the number of primordial, primary, secondary, and Graafian follicles and the number of corpus luteum.

Parameters	WAS F(1,24)	*p*	ηp^2^	CoQ10 F(1,24)	*p*	ηp^2^	WAS × CoQ10 F(1,24)	*p*	ηp^2^
Primordial follicle	**5.327**	**0.030**	**0.182**	3.128	0.090	0.115	0.009	0.927	0.000
Primary follicle	0.978	0.333	0.039	1.441	0.242	0.057	4.158	0.053	0.148
Secondary follicle	0.011	0.917	0.000	0.159	0.694	0.007	0.278	0.603	0.011
Graafian follicle	1.292	0.267	0.051	1.530	0.228	0.060	0.541	0.469	0.022
Corpus luteum	2.793	0.108	0.104	0.747	0.396	0.030	0.087	0.771	0.004

Data are presented as F values, *p* values, and partial eta squared (ηp^2^) obtained from two-way ANOVA. Bold values indicate statistical significance at *p* < 0.05.

**Table 6:** Two-way ANOVA results showing the main effects of WAS and CoQ10 and their interaction on the germinative epithelium thickness, angiogenesis histoscore, mast cell count, and VEGF histoscore.

Parameters	WAS F(1,24)	*p*	ηp^2^	CoQ10 F(1,24)	*p*	ηp^2^	WAS × CoQ10 F(1,24)	*p*	ηp^2^
Germinative epithelium thickness	**6.552**	**0.017**	0.214	**35.196**	**<0.001**	**0.595**	2.003	0.170	0.077
Angiogenesis histoscore	0.429	0.519	0.018	3.857	0.061	0.138	**6.857**	**0.015**	**0.222**
Mast cell count	0.183	0.673	0.008	0.244	0.626	0.010	2.600	0.120	0.098
VEGF histoscore	**5.115**	**0.033**	0.176	0.272	0.607	0.011	0.272	0.607	0.011

Data are presented as F values, *p* values, and partial eta squared (ηp^2^) obtained from two-way ANOVA. Bold values indicate statistical significance at *p* < 0.05.

## Data Availability

All data generated and/or analyzed during the current study are included in this paper and can be obtained from the corresponding author upon reasonable request.

## Abbreviations

The following abbreviations are used in this manuscript:

ANOVA	Analysis of variance
CoQ10	Coenzyme Q10
CUMS	Chronic unpredictable mild stress
ELISA	Enzyme-linked immunosorbent assay
E2	17-β estradiol
GnRH	Gonadotropin-releasing hormone
H&E	Hematoxylin and eosin
HPA	Hypothalamus–pituitary–adrenal
HPO	Hypothalamus pituitary ovarian
IHC	Immunohistochemistry
MCs	Mast cells
OHSS	Ovarian hyperstimulation syndrome
OSE	Ovarian surface epithelium
OVX	Ovariectomized
8-OHdG	8-hydroxy-deoxyguanosine
RT-qPCR	Quantitative real-time PCR
PAS	Periodic acid–Schiff
RW	Relative organ weight
SEM	Standard error of the mean
SIM	Sexual incentive motivation
TWM	Time spent near the male rat
TWF	Time spent near the female rat
TST	Total social time
TIM	Duration of investigation directed toward the male rat
TIF	Duration of investigation directed toward the female rat
TIT	Total investigation time
WAS	Water avoidance stress
T-AOC	Total antioxidant capacity
VEGF	Vascular endothelial growth factor
ηp^2^	Partial eta-squared
